# Rechallenge with EGFR-TKI after failure of immunotherapy is considered an effective treatment for advanced lung adenocarcinoma patients with EGFR exon 19 deletion: a case report

**DOI:** 10.3389/fmed.2023.1168220

**Published:** 2023-06-29

**Authors:** Shubin Chen, Qitao Yu, Wei Jiang, Yukun Lu, Yun Zhao, Huilin Wang

**Affiliations:** ^1^Medical Oncology of Respiratory, Guangxi Cancer Hospital, Guangxi Medical University Cancer Hospital, Nanning, Guangxi, China; ^2^Department of Pathology, Guangxi Cancer Hospital, Guangxi Medical University Cancer Hospital, Nanning, Guangxi, China

**Keywords:** advanced lung adenocarcinoma, EGFR exon 19 deletion, immunotherapy, EGFR-TKI, rechallenge

## Abstract

In advanced lung adenocarcinoma patients with epidermal growth factor receptor (EGFR) mutation, epidermal growth factor receptor-tyrosine kinase inhibitors (EGFR-TKIs) have an excellent and long-lasting therapeutic response; however, virtually all patients eventually develop drug resistance and experience disease progression. The use of immunotherapy after EGFR-TKIs may be a successful therapeutic option for individuals who are resistant to them. It is still unclear if EGFR-TKIs can be administered again after immunotherapy has failed. We describe a case of a 37-year-old woman who was found to have T4N3M1a stage IVa lung adenocarcinoma. Amplification refractory mutation system PCR (ARMS-PCR) genetic testing suggested EGFR exon 19 deletion. The patient was initially treated with a regimen of icotinib (125  mg tid) combined with anlotinib (8  mg qd d1-d14) with an optimal efficacy rating of partial response (PR) and was granted a PFS of 7  months. In second-line treatment, the patient received three cycles of a KN046 (KN046 is a bispecific antibody inhibitor of PD-L1 and CTLA-4) 295  mg d1, pemetrexed 800 mg d1, plus carboplatin 750  mg d1 regimen, with an optimal efficacy rating of stable disease (SD) on CT. The third-line therapy was chosen to be afatinib with docetaxel, and the patient was evaluated for PR on CT. Up to 15 August 2022, the patient had a progression free survival (PFS) of 14 months. The successful treatment of this patient is a reminder that EGFR-TKI rechallenge in EGFR exon 19 deletion patients with EGFR-TKI resistance, in which immunotherapy has failed, may be effective.

## Introduction

Epidermal growth factor receptor (EGFR) mutations, the most frequent genetic drivers of advanced lung cancer, have a 50% likelihood of occurring in Asian populations, and targeted treatment has rapidly improved over the past 10 years (1). The most prevalent EGFR mutation is the exon 19 deletion (2). EGFR-TKIs block the autophosphorylation of EGFR receptor tyrosine, which further blocks downstream signaling and limits EGFR-dependent cell growth, having an anti-tumor impact. Although advanced lung adenocarcinoma patients with EGFR mutations now have a significantly better prognosis thanks to epidermal growth factor receptor tyrosine kinase inhibitors (EGFR-TKIs), tumors will always acquire medication resistance for a variety of reasons.

The popularity of immune checkpoint inhibitors (ICIs) in the management of advanced non-small cell lung cancer has grown significantly. However, it was unclear from the existing research whether immune checkpoint inhibitors are appropriate for individuals with EGFR mutations. According to certain studies, immune checkpoint inhibitors can cause serious toxic side effects in individuals with EGFR mutations and potential fulminant disease progression (3–5). In contrast, a subgroup analysis of IMPOWER150 revealed that patients with EGFR mutations responded favorably to an atezolizumab, bevacizumab, carboplatin, plus paclitaxel regimen (6).

Several findings regarding the therapeutic effectiveness of EGFR-TKIs rechallenge in EGFR-mutant non-small cell lung cancer (NSCLC) have recently been published (7–9). However, the question of whether patients who have failed EGFR-TKI therapy with an immune checkpoint inhibitor for second-line treatment can be re-challenged with EGFR-TKI following another bout of disease progression lacks a somewhat conclusive response. In this case, we describe the effectiveness of EGFR-TKI rechallenge in a patient with advanced NSCLC with EGFR exon 19 deletion who was treated with an immune checkpoint inhibitor for second-line treatment and recurrence of disease progression.

## Case presentation

**Figure 1:**
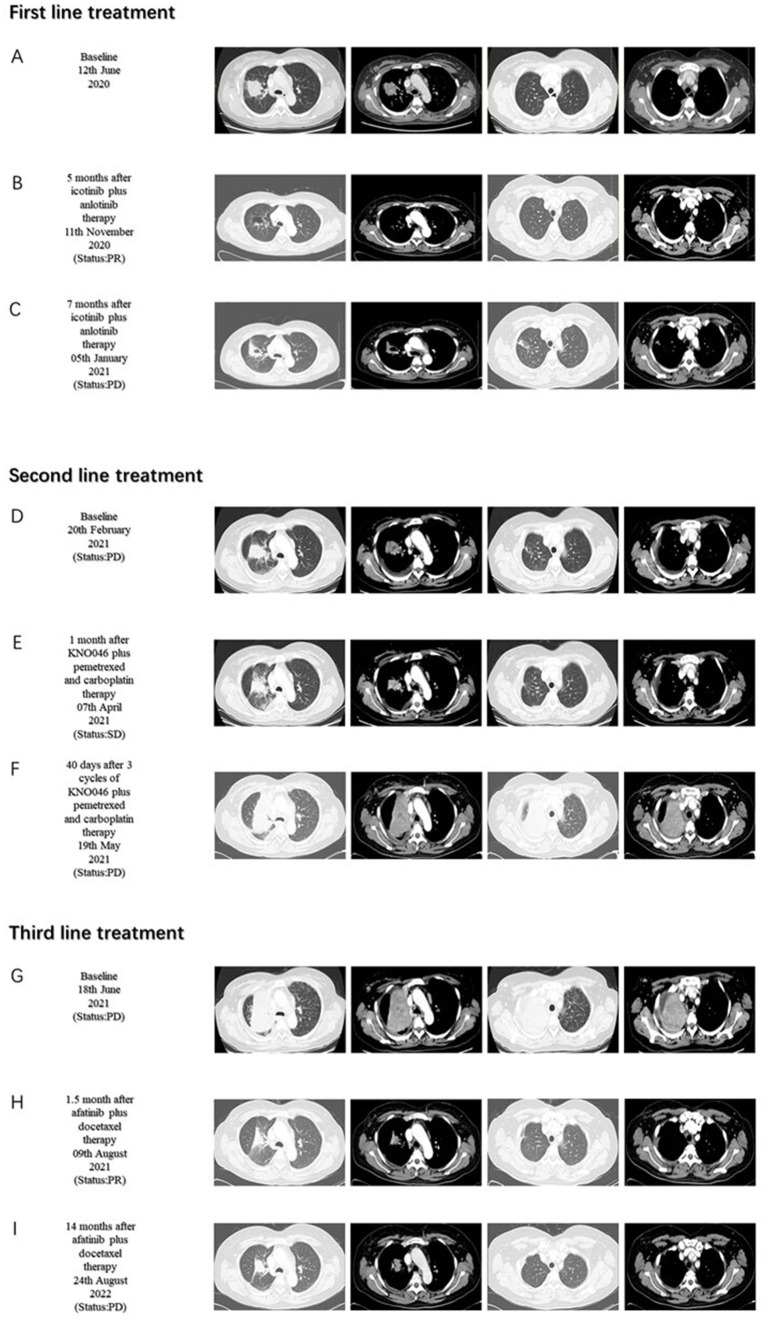
Chest computed tomography (CT) scans before and after treatment. First-line treatment baseline **(A)**. Five months after icotinib plus anlotinib therapy **(B)**. Seven months after icotinib plus anlotinib therapy **(C)**. Second-line treatment baseline **(D)**. One month after KNO046 plus pemetrexed and carboplatin therapy **(E)**. Forty days after three cycles of KNO046 plus pemetrexed and carboplatin therapy **(F)**. Third-line treatment baseline **(G)**. 1.5 month after afatinib plus docetaxel therapy **(H)**. Fourteen months after afatinib plus docetaxel therapy **(I)**.

**Figure 2:**
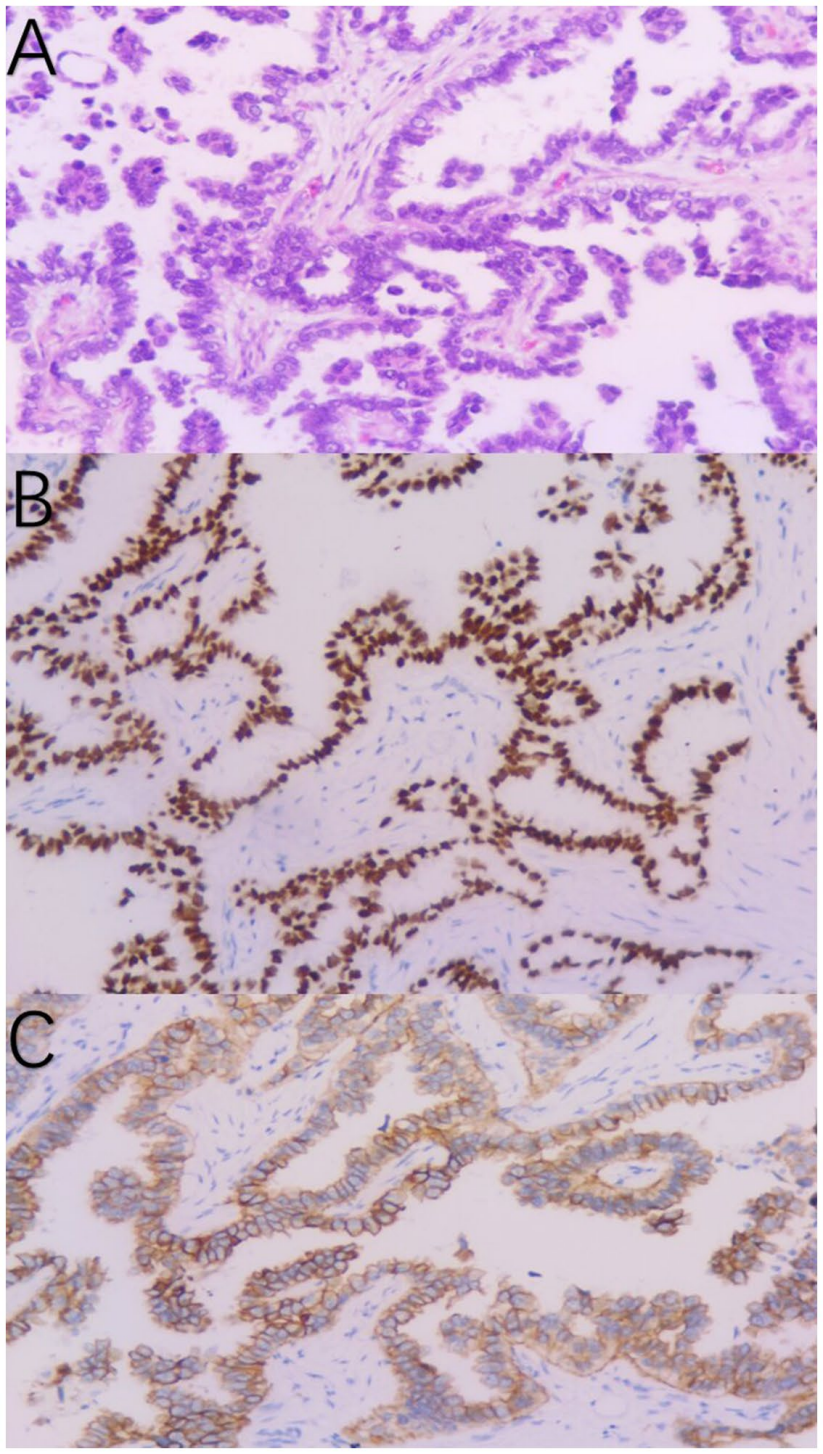
Pathological diagnosis of the right supraclavicular lymph node biopsy **(A)**. Immunohistochemistry staining: TTF-1 **(B)** and CK7 **(C)** were positive, consistent with adenocarcinoma of pulmonary origin.

**Figure 3:**
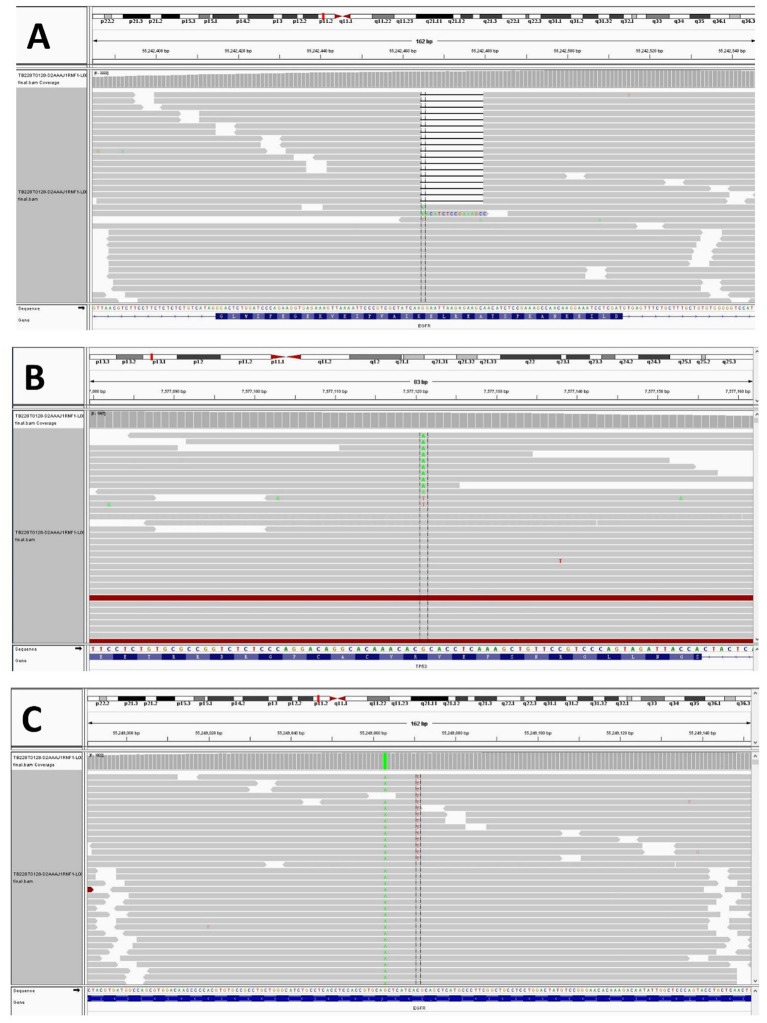
NGS test result showing the EGFR exon 19 p.E746_A750del **(A)**, TP53 exon 8 p.R273C **(B)**, and EGFR exon 20 p.T790M **(C)**.

**Figure 4:**
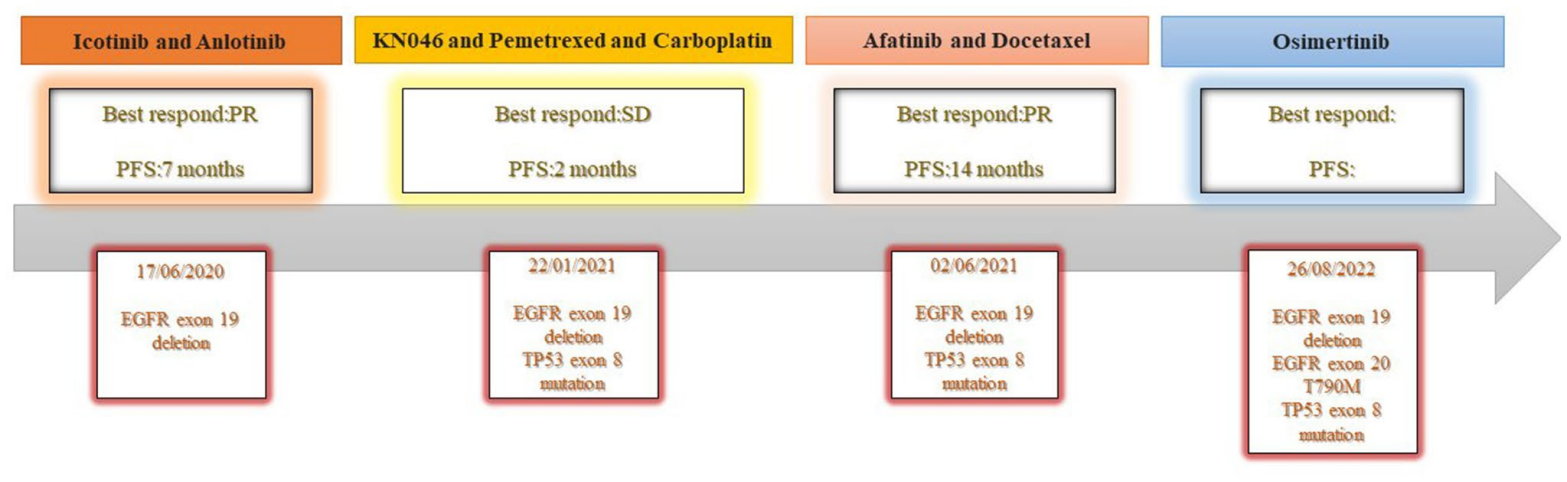
Timeline of the treatment of the patient.

A 37-year-old woman was hospitalized after an occupying lung lesion and an enlarged right supraclavicular lymph node were discovered. A computed tomography (CT) scan revealed several lymph node metastases in the right supraclavicular area, mediastinum, and lung, in addition to a mass in the upper lobe of the right lung, right pleural metastasis and multiple nodules in both lungs, with a high probability of inflammatory lesions combined with some metastases (Figure 1A). There were no lesions seen on the brain’s magnetic resonance imaging (MRI). No bone metastases were detected by bone imaging. A right supraclavicular lymph node biopsy revealed adenocarcinoma and CK7(+), TTF-1(+), and P40(−) immunohistochemistry results were consistent with adenocarcinoma of pulmonary origin (Figure 2). The patient was found to have T4N3M1a stage IVa lung adenocarcinoma. Amplification refractory mutation system PCR (ARMS-PCR) genetic testing suggested EGFR exon 19 deletion, with no detectable aberrant changes in the ALK, ROS1, KRAS, BRAF, RET, NRAS, PIK3CA, and HER2 genes. We advocated that the patient engaged in a clinical trial using both gefitinib and anlotinib, but for a variety of reasons, the patient opted not to do so. However, the patient requested anlotinib therapy; icotinib 125 mg tid and anlotinib 8 mg qd were chosen as the patient’s initial treatment regimen. According to the response evaluation criteria for solid tumors (RECIST v1.1), significant lung tumor lesions decreased and developed cavity-like alterations after 5 months of therapy, demonstrating partial response (PR) (Figure 1B). Additionally, there was a decline in the serum tumor biomarkers CA199, CA153, CA125, and CEA. The upper lobe of the right lung had a noticeably larger tumor lesion on 5 January 2021, which was classified as progressing disease (PD) using the RECIST v1.1 assessment criteria (Figure 1C). The patient underwent CT-guided biopsy of lung lesions by puncture on 8 January 2021. Immunohistochemistry showed adenocarcinoma, TTF-1 (+), CK7 (+), P40 (−), and Ki67 (+20%). Further blood and tumor specimens from the patient were subjected to next-generation sequencing (NGS) testing, showing EGFR exon 19 deletion (Figure 3A) with an abundance of 2.4% in tumor specimens and TP53 exon 8 mutation (Figure 3B) with an abundance of 0.9% in tumor specimens. Programmed cell death-Ligand protein 1(PD-L1) expression was also examined, and the result suggested that the PD-L1 tumor cell proportion score (TPS) was <1%. The patient opted to participate in a clinical study as the second line of treatment, receiving three cycles of KN046 (a bispecific antibody inhibitor of PD-L1 and CTLA-4) with pemetrexed 800 mg/d1 and carboplatin 750 mg/d1, with an effectiveness rating of SD on CT scan (Figures 1D,E). Unfortunately, the patient subsequently ceased the treatment because of grade 3 hepatitis brought on by immunotherapy. Forty days after discontinuation of the regimen, the patient was evaluated as PD (Figure 1F), with a significant increase in serum tumor biomarkers. After that, the patient underwent fiberoptic bronchoscopy, and further lung lesions were sampled. Repeated NGS analysis of the patient’s blood and tumor samples revealed an EGFR exon 19 deletion with an abundance of 41.9% in tumor specimens, suggesting a potential re-sensitization to EGFR-TKI. Because immune checkpoint inhibitors interact with EGFR-TKI to produce serious adverse effects, we restarted EGFR-TKI therapy after 3 months of discontinuation of immune checkpoint inhibitors based on the elution time of the immune checkpoint inhibitors. Third-line therapy involved the use of afatinib 40 mg every day in conjunction with docetaxel 110 mg every 3 weeks (Figure 1G). On CT, the patient was assessed for a partial response after two cycles of therapy (Figure 1H), with no drug-related adverse events and a decrease in serum tumor biomarkers once again. On 15 August 2022, the patient underwent a CT examination which revealed a significant re-enlargement of the tumour and was considered to be drug resistant again, getting a progression free survival (PFS) of 14 months (Figure 1I). A repeat CT-guided puncture biopsy of the lung lesion and repeat NGS testing of the patient’s blood and tumor specimen suggested the presence of a T790M mutation in exon 20 of EGFR (Figure 3C). The patient is currently being treated with osimertinib (Osimertinib, a third-generation EGFR-TKI that targets EGFR mutations and T790M-resistant mutations and inhibits tyrosine kinase phosphorylation by creating a covalent bond with the C797 residue at the ATP binding site of EGFR mutations and T790M mutations, acting as a tumor suppressor). On 11 March 2023, the patient underwent a CT examination that revealed a significant shrinking of the tumor, considered to be a partial response. The timeline of the treatment of the patient is presented in Figure 4.

## Discussion

With the publication of the IPASS study results (10), EGFR-TKIs have officially become the treatment of choice for patients with EGFR mutations. EGFR-TKIs have startlingly high effectiveness; however, tumors will inevitably acquire therapeutic resistance. The results of several clinical trials (11–13) show that immunotherapy may also be an effective therapeutic option for individuals with EGFR mutations, even though the effectiveness of immunotherapy for patients with EGFR mutations is currently unclear. The feasibility of resuming EGFR-TKIs therapy for these patients after immunotherapy has failed is still a hotly contested issue.

In our case report, we described the treatment of a patient with advanced lung adenocarcinoma who had an EGFR exon 19 deletion mutation. The above patient received first-line treatment with a first-generation EGFR-TKI in conjunction with anti-angiogenic medications and was granted a PFS of 7 months as a result. Immunotherapy was discontinued after a short course due to immunotherapy-related adverse effects. Subsequently, the patient received second-generation EGFR-TKI in combination with chemotherapy. The rationale for the program was based on the good efficacy reported by NEJ009 (14), demonstrating 14 months PFS with a partial response, showing that TKI combination chemotherapy had good anti-tumor activity against EGFR mutation combined with TP53 mutation. This example serves as proof that treating these patients sequentially with immune checkpoint inhibitors after EGFR-TKI treatment has failed, followed by an EGFR-TKI rechallenge, may improve their therapeutic effectiveness.

We searched for EGFR-TKI rechallenge following immunotherapy failure in the literature. In a study byKaira et al. (15), a retrospective analysis of 75 patients with advanced NSCLC and sensitive EGFR mutations revealed that approximately half of the patients had a good response to EGFR-TKI rechallenge, while approximately 25% of all patients had no response to EGFR-TKI rechallenge following the failure of immunotherapy. In the study, two cases were reported in which a dramatic response upon EGFR-TKI rechallenge was achieved. Nivolumab was started in Case 1 in a 64-year-old woman with advanced lung cancer since EGFR-TKI and systemic chemotherapy were ineffective. One month after its administration, PD was observed; therefore, the patient was treated with afatinib rechallenge. Two weeks after afatinib treatment, a drastic response of tumor shrinkage in multiple pulmonary metastases was identified, with an eventual PFS of 10 months. In Case 2, a 39-year-old woman with lung adenocarcinoma underwent multiple treatments with platinum-based or second or third generation EGFR-TKIs regimens; however, the patient developed therapeutic resistance. Nivolumab was started as a result, but a month after it was administered, indication of considerable growth of many brain metastases was evident. The brain metastases nearly completely vanished after 1 month of treatment with erlotinib and bevacizumab, leading to a PFS of 9 months. For NSCLC patients who have developed resistance to EGFR-TKIs, EGFR-TKI rechallenge after immunotherapy treatment may be an effective therapeutic option.

The underlying mechanisms through which EGFR-TKIs rechallenge immediately after the preceding immunotherapy is effective in patients with EGFR-TKIs resistance is unknown. Previous research has demonstrated that the expression of PD-L1 changes throughout the use of EGFR-TKIs, with certain patients showing a tendency to upregulate its expression after developing resistance to EGFR-TKIs (16–18). Peng et al. proposed that EGFR-TKI resistance promotes immune escape in lung cancer via increased PD-L1 expression; increased PD-L1 expression after EGFR-TKI resistance impairs lymphocyte activation and cytotoxicity *in vitro* and *in vivo*, whereas PD-L1 downregulation partially restores lymphocyte cytotoxicity (19). The idea is that PD-1/PD-L1 blocking therapy may restore EGFR-TKI sensitivity in patients with malignancies that are resistant to treatment. According to Sugiyama et al. (20), the use of EGFR-TKI in conjunction with PD-1 inhibitor may be preferable since it affects the function of regulatory T cells in the tumor microenvironment. The synergistic impact of EGFR-TKI and PD-1 inhibitor in the setting of long-term EGFR-TKI treatment may restore the anti-tumor activity. According to studies (21, 22), patients’ tumor mutation burden (TMB) increases after receiving EGFR-TKI treatment. This increased TMB has a negative correlation with the response to EGFR TKIs (21). Patient’s TMB declines after a certain period of immune checkpoint blockade therapy, and the EGFR-TKI may be reverted to show sensitivity to the tumor. Jia et al. investigated the immunological environment of lung cancer tumor cells that showed a strong early reaction to EGFR-TKI. The authors discovered a short-term increase in CD8+ T cells during the initiation of EGFR-TKI treatment, indicating that CD8+ T cell activity may impact EGFR-TKI effectiveness (23). We have reason to assume that PD-1/PD-L1 inhibition, which restores the initial activity of CD8+ T cells, will also restore the activity of EGFR-TKI. Further research on this potential follow-up therapy should be pursued, even if it is complicated to pinpoint the precise mechanism by which sensitivity to EGFR-TKIs is immediately restored following immune checkpoint blockade treatment.

As a whole, the following aspects standout in our case: in order to determine the patient’s potential medication resistance mechanisms, a second biopsy was taken after the disease progressed in the patient. Secondly, the patient developed immune-related hepatitis after using immunotherapy, which persisted after stopping immunotherapy. Aafatinib is not hepatically metabolized, and it has the potential to exacerbate liver damage if other EGFR-TKIs were used. Finally, investigations on the EGFR mutation in conjunction with TP53 mutation are ongoing, and our novel application of TKI in combination with chemotherapy has resulted in a longer PFS for the patient. Despite the significance of the case’s findings, there is one drawback: osimertinib was not selected as the first-line therapy in this instance.

The successful treatment of this patient serves as a reminder that EGFR-TKI rechallenge in EGFR exon 19 deletion patients with EGFR resistance, in which immunotherapy has failed, may be effective. A sizable prospective clinical study may be required to further corroborate the case’s conclusion.

## Data availability statement

The original contributions presented in the study are included in the article/supplementary material, further inquiries can be directed to the corresponding author.

## Ethics statement

The studies involving human participants were reviewed and approved by Guangxi Medical University Affiliated Cancer Hospital’s Ethical Committee. The patients/participants provided their written informed consent to participate in this study. Written informed consent was obtained from the individual(s) for the publication of any potentially identifiable images or data included in this article.

## Author contributions

SC and QY wrote the manuscript. WJ provided constructive discussion and revised the paper. YL and YZ provided the pathology and CT images. HW obtained informed consent from the patient, administered treatment to the patient, and provided the clinical data of the patient. All authors contributed to the article and approved the submitted version.

## Conflict of interest

The authors declare that the research was conducted in the absence of any commercial or financial relationships that could be construed as a potential conflict of interest.

## Publisher’s note

All claims expressed in this article are solely those of the authors and do not necessarily represent those of their affiliated organizations, or those of the publisher, the editors and the reviewers. Any product that may be evaluated in this article, or claim that may be made by its manufacturer, is not guaranteed or endorsed by the publisher.
